# High Level of Inflammatory Cytokines in the Tears: A Bridge of Patients with Concomitant Exotropia and Dry Eye

**DOI:** 10.1155/2021/5662550

**Published:** 2021-10-07

**Authors:** Feng Gao, Xiaoping Hong, Fadian Ding, Shirong Huang, Wei Lian, Hanjun Wang, Weidong Zheng, Jun Ni, Min Chen, Qicai Liu

**Affiliations:** ^1^Department of Pathology, 1st Affiliated Hospital, Fujian Medical University, Fuzhou 350004, China; ^2^Department of Ophthalmology, The Second Affiliated Hospital, Fujian Medical University, Quanzhou 362000, China; ^3^Department of Ophthalmology, 1st Affiliated Hospital, Fujian Medical University, Fuzhou 350004, China; ^4^Department of Surgery, 1st Affiliated Hospital, Fujian Medical University, Fuzhou 350004, China; ^5^Department of Laboratory Medicine, Fujian Medical University, Fuzhou 350004, China; ^6^Department of Rehabilitation Medicine, 1st Affiliated Hospital, Fujian Medical University, Fuzhou 350004, China; ^7^Center for Reproductive Medicine, 1st Affiliated Hospital, Fujian Medical University, Fuzhou 350004, China

## Abstract

Concomitant exotropia have obvious symptoms of eye discomfort in adults, and the presence of ocular surface inflammation in patients may be important mediators between concomitant exotropia and dry eye. Oculus Keratograph eye comprehensive analyzer was performed to detect noninvasive tear break time, noninvasive tear height, and eye red index, while the ocular surface disease index and schirmer I testing were made. The levels of IL-6, IL-10, IL-17A, IL-12P70, INF-*γ*, and TNF-*α* were detected in tears in patients with concomitant exotropia and healthy controls matched by age and gender through the Simoa technology. IL-6 was significantly higher in patients with concomitant exotropia (4.683 ± 1.329) pg/mL than that in normal group (1.455 ± 0.391) pg/mL, *p* = 0.0304. TNF-*α* was also significantly higher in patients (0.2095 ± 0.0703) pg/mL than normal group (0.0513 ± 0.0149) pg/mL, *p* = 0.0397. The levels of inflammatory factors in strabismic patients vs. normal controls were as follows: IL-17A (0.1551 pg/mL︰0.0793 pg/mL), IL-10 (0.3358 pg/mL︰0.0513 pg/mL), IL-12p70 (0.0253 pg/mL︰0.0099 pg/mL), and INF-*γ* (0.0284 pg/mL︰0.009 pg/mL) were detected, and the median of them in concomitant strabismus was 1.96-6.55-fold as much as the control group. High levels of inflammatory cytokines in tears of patients with concomitant exotropia, which may be a potentially factor promoted the occurrence of dry eye in the patients with concomitant exotropia.

## 1. Introduction

Strabismus is a disease of eye deviation caused by the imbalance of external eye muscles in both eyes. When one eye fixates on a target, the direction of the other eye axis deviates from the direction of fixation. The normal cornea is in a central position due to prolonged oblique or alternate binoculars in one eye, which can lead to corneal overexposure and tear film instability. Previous study had found that the height of the river of tears is reduced and the tear film is unstable, and the eye red index is increased in concomitant exotropia [1].

Giannaccare et al. hypothesized that long-term deviation of the eyeball from its primary location could lead to chronic changes in the ocular surface, which could result in dry eyes through two possible mechanisms [2]. Firstly, increased exposure area of conjunctiva may lead to thinner tear film lipid layer and increased tear film instability [2]. Secondly, abnormal anatomical and functional relationships between the eyelids and the eyeball increase mechanical friction to the conjunctival epithelium, leading to microtrauma associated with blinking [2]. Mechanical damage to the conjunctival epithelium caused by tear film instability further activates a series of inflammatory events, and long-term inflammatory cell infiltration and increased expression of inflammatory factors lead to further damage to the ocular surface system. We have therefore investigated if there are differences about inflammatory cytokines in our cohort of patients with concomitant exotropia and control group.

## 2. Methods and Materials

### 2.1. Participants and Clinical Information Collection

We included sixty-six patients with concomitant exotropia and sixty-six controls; inclusion criteria: (1) concomitant exotropia diagnosed in outpatient department as meeting surgical criteria; (2) the first oblique viewing angle is equal to the second oblique viewing angle, and there is no ability to control the anteroposterion in the far or near viewing, and the horizontal oblique viewing degree is >15PD (prism degree); (3) no combination of dissociated vertical deviation (DVD), superior oblique muscle palsy (SOP), and other diseases, vertical strabismus < 5PD; (4) no complaints of eye discomfort except strabismus; (5) no history of ophthalmic disease or operation; (6) patients with paralytic strabismus, secondary strabismus, perceptual strabismus, residual strabismus, combined keratitis, eyelid gland dysfunction, and Sjogren's syndrome were excluded. There was no significant difference in the number of cases, strabismus, and dominant eye among all subgroups, which showed comparability. There were no significant difference in the age or gender ratios of participants between the concomitant exotropia group and control group (Table 1). At the same time, we collected the ocular surface disease index (OSDI), the height of the river of tears, the tear film rupture time, the tear secretion level, and the eye red index including the adults and children.

### 2.2. Ocular Surface Analysis

Oculus Keratograph eye comprehensive analyzer was performed to detect noninvasive tear break time (NIBUT), noninvasive tear height (NITMH), and eye red index. Both eyes of exotropia patients were measured separately. First, the right eye was measured, then the left eye was measured for 3 consecutive times, and the average value was taken. All patients were examined by the same experienced staff [3]. The normal reference value of tear film rupture time is 10-15 mm, while <10 mm is hyposecretion, and <5 mm is dry eye. The height of river of tears was 0.2 mm as the critical value, and <0.2 mm was diagnosed as dry eye [4]. The eye red index was less than 2 points for mild hyperemia, 2.1 to 3.0 points for moderate hyperemia, and 3.1 to 4.0 points for severe hyperemia.

### 2.3. Schirmer I Testing and OSDI

To obtain the height of the river of tears at the lowest part of the crescent curve page for measurement, the unit is expressed in mm, and the critical value of the height of the river of tears is 0.2 mm[5]. The normal reference value of Schirmer I testing is 10-15 mm/5 min, while <10 mm/5 min is hyposecretion, and <5 mm/5 min is dry eye. OSDI test includes 12 questions and three parts: part 1 refers to ocular pain or visual difficulties; part 2 is about visual functionality; and part 3 is analyses of environmental factors.

### 2.4. Tears Collect

Inclusion criteria for the experimental group: (1) clinical diagnosis was concomitant exotropia, and refractive errors (spherical ≤ ±1.00D, anastigmate or cylindrical ≤ 0.50) were corrected by wearing glasses, while there were no neurological diseases or craniofacial abnormalities. (2) Except for strabismus and refractive errors, the patients had no history of other visual defects and eye diseases and no family genetic history (including history of strabismus). Inclusion criteria for the control group: (1) refractive errors (spherical ≤ ±1.00D, anastigmate or cylindrical ≤ 0.50) were corrected by wearing glasses while no neurological disease, no craniofacial abnormalities, no history of visual impairment or eye disease, and no family history of hereditary diseases (including strabismus). (2) No related symptoms such as dry eyes. We collected relevant clinical information, including gender, age, and course of the disease, corneal light reflection test, eye movement (intermittence/constancy), strabismus degree, and the dominant eye. There were no significant differences in the age or gender ratios of participants between the concomitant exotropia group and control group (Table 2). The study was approved by the ethics committee of 1st Affiliated Hospital of Fujian Medical University. Informed consent was obtained from legal guardians or participants.

We use tears adsorption filter paper (Tianjin Jingming New Technology Development Co., LTD., Tianjin; China Strip, hereinafter referred to as China Strip) to collect tears from the subjects. Schirmer tear test strips were customised with filter paper labeled with fluorescent-sodium and are of structure of 40 mm × 5 mm filter strips with millimeter scale ranging from 0 to 30 mm. The tears samples were eluted with 200 μL PBS and shaken for 5 hours at room temperature, and the samples were stored in a refrigerator at -80°C [6].

### 2.5. Measurement of Inflammatory Cytokines in the Tears

For SIMOA experiments, tear samples were prepared according to the kit specific manufacturer's instruction. Inflammatory cytokine levels were measured using SIMOA HD-X Cytokine 6-Plex panel standards, and samples were run utilizing manufacturer's assay instructions.

### 2.6. Statistical Analyses

All of the data was analyzed with the SPSS 20.0 program (IBM Corp. SPSS Statistics for Windows, version 20.0, Armonk, NY, USA). All the experimental results were statistically analyzed to compare the differences between groups by *t*-test, and *p* < 0.05 was considered statistically significant.

## 3. Results

### 3.1. Dry Eye-Related Indicator Is Abnormal in Adults While It Is Normal in Children

In order to further clarify the ocular surface of patients with concomitant exotropia. The ocular surface disease index (OSDI), the height of the river of tears, the tear film rupture time, the Schirmer I testing, and the eye red index were measured in 66 patients with concomitant exotropia including adults and children. In our study, we found that the height of the river of tears was decreased (0.2061 ± 0.0068 mm vs. 0.2353 ± 0.0083 mm, *p* < 0.01). And the eye red index of adult patients with exotropia was increased compared to the control group (1.497 ± 0.1348 vs. 1.161 ± 0.1008, *p* < 0.05). Schirmer I testing showed that the tear secretion level of adult patients with exotropia was lower than that of control group (9.333 ± 0.5633 mm/5 min vs. 10.84 ± 0.4815 mm/5 min, *p* < 0.05). The OSDI score (18.99 ± 2.426 vs. 14.58 ± 2.101, *p* = 0.1735) and tear film rupture time (6.881 ± 0.7390 s vs. 8.846 ± 0.7674 s, *p* = 0.069) were no found the statistical difference between concomitant exotropia and control.

**Figure 1:**
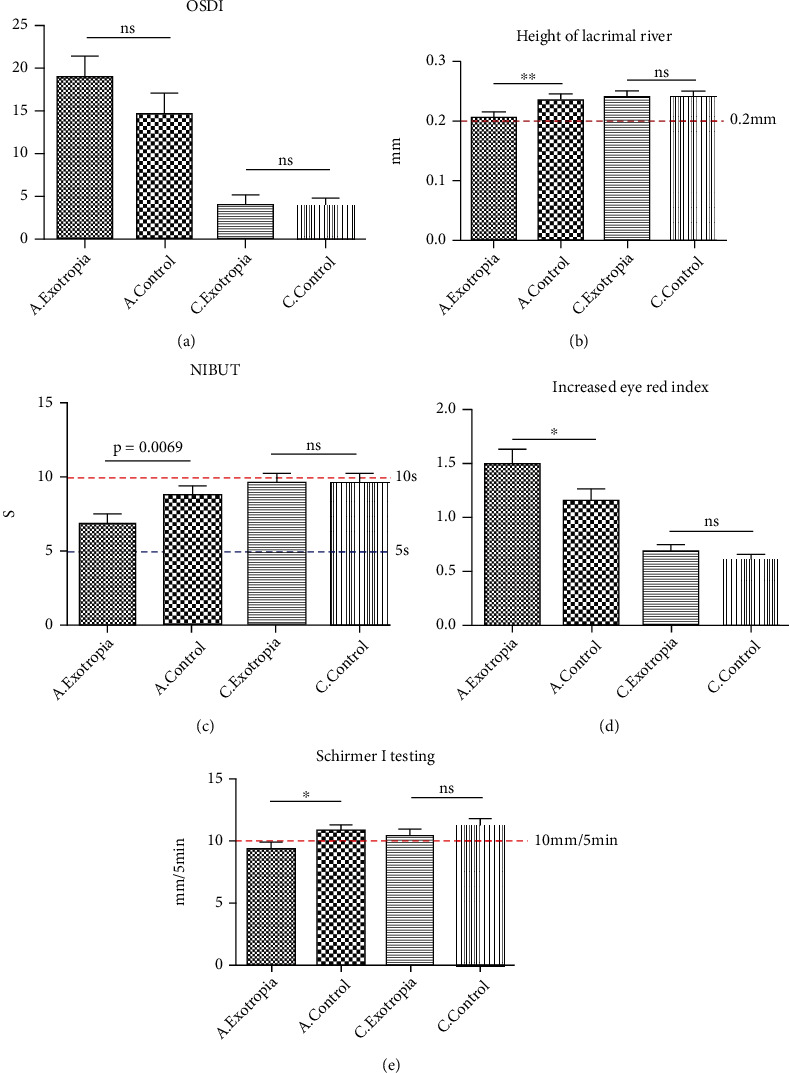
The ocular surface analysis in patients with concomitant exotropia. (a) The ocular surface disease index (OSDI); (b) the height of the river of tears; (c) the noninvasive tear film rupture time (NIBUT); (d) the eye red index; (e): the schirmer I testing. A. exotropia: concomitant exotropia in adult; C. exotropia: concomitant exotropia in children; A. control: control group in adult; C. control: control group children.

However, in the children group, the measurement of OSDI (4.080 ± 0.9179 vs. 3.837 ± 0.9062, *p* > 0.05), tear film rupture time (9.658 ± 0.7309 s vs. 9.728 ± 0.8429 s, *p* > 0.05), eye red index (0.6833 ± 0.06380 vs. 0.6000 ± 0.05296, *p* > 0.05), river of tears height (0.2410 ± 0.0060 mm vs. 0.2417 ± 0.0069 mm, *p* > 0.05), and tear secretion level (10.42 ± 0.4133 mm/5 min vs. 11.31 ± 0.4016 mm/5 min, *p* > 0.05) did not find statistical differences (Figure 1).

### 3.2. Inflammation Cytokines in Tears Promoted the Occurrence of Dry Eye in Children with Concomitant Exotropia

**Figure 2:**
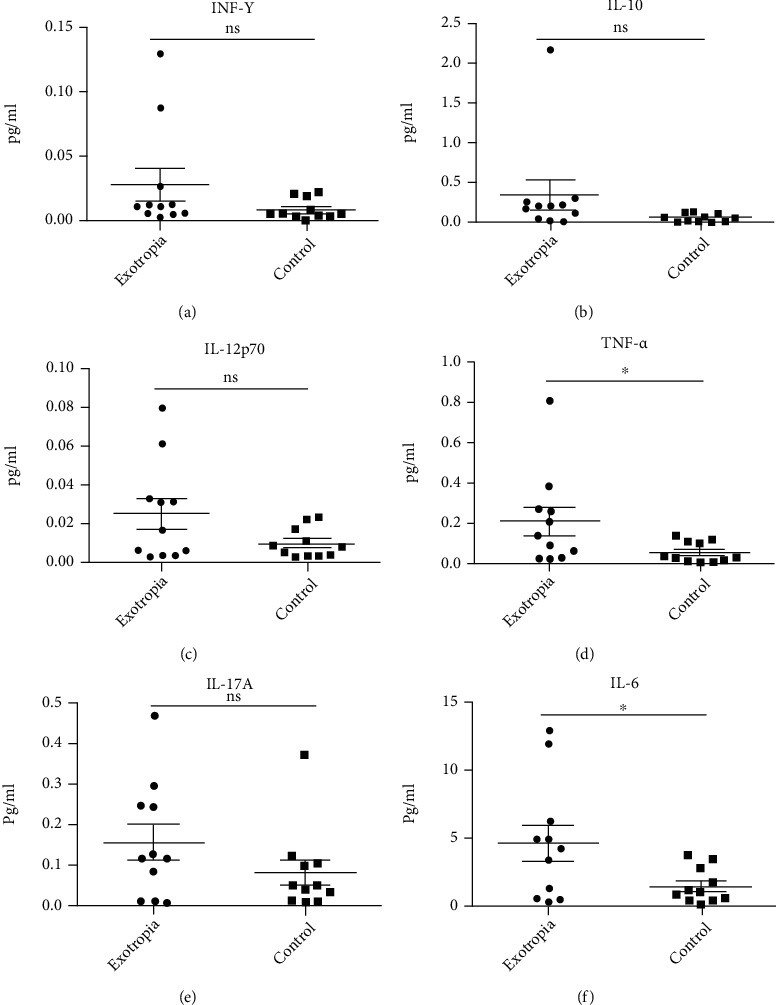
The level of inflammatory cytokines in tears of patients with concomitant exotropia. (a) INF-*γ*; (b) IL-10; (c) IL-12p70; (d) TNF-*α*; (e) IL-17A; (f) IL-6.

We found that adults had obvious dry eye-related symptoms in concomitant exotropia, while children had no significant difference compared to the control group. A variety of tear proteins are significantly associated with aging in strabismus patients, many of which were associated with inflammation, immune response, and cell death [7]. The persistence of inflammatory cytokines in tears may be associated with the occurrence of dry eyes in concomitant exotropia. To investigate the different levels in the children with exotropia and control, we used SIMOA technology to measure the levels of inflammatory cytokines in tears in strabismus and normal patients. Levels of INF-*γ*, IL-10, IL-12p70, IL-17A, IL-6, and TNF-*α* were measured. We found the expression of INF-*γ*, IL-10, IL-12p70, and IL-17A in tears of strabismus patients, which were no significantly higher than those of normal subjects, while IL-6 and TNF-*α* were statistically significant (Table 3, Figure 2).

### 3.3. Mechanistic Hypothesis of Ocular Surface Inflammation Cytokines and Dry Eye in Patients with Concomitant Exotropia

**Figure 3:**
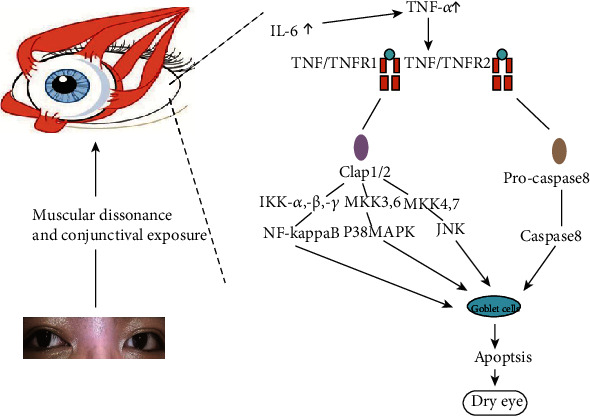
The role of TNF-*α* and IL-10 in the ocular inflammation and dry eyes.

The role of multiple inflammatory factors in dry eye symptoms of patients with concomitant exotropia is unknown. Firstly, increased exposure and local lesions in concomitant exotropia lead to increased expression of inflammatory factors, especially IL-6 and TNF-*α*, in the tears. Secondly, the increased expression of inflammatory cytokines may lead to goblet cell apoptosis and the occurrence of dry eye. Thirdly, related literature supports that IL-6 and TNF-*α* may activate downstream NF-*κ*B [8], p38MAPK [9], and JNK[10] pathways through TNF receptor 1 and activate downstream caspase-8[11] through TNF receptor 2 to induce apoptosis of functional cells (Figure 3).

## 4. Discussion

We have shown here that dry eye-related indicator is abnormal in adults while it is normal in children, which included the ocular surface disease index (OSDI), the height of the river of tears, the tear film rupture time, the tear secretion level, and the eye red index. The concentration of tear mediators increased obviously in concomitant exotropia, and the IL-6 and TNF-*α* could be important mediators of inflammation. Inflammatory cytokines may be involved in goblet cell apoptosis through a variety of signaling pathways. Early intervention in these patients can avoid long-term high concentration of inflammatory factors and the occurrence of dry eye.

Patients with concomitant exotropia have dry eyes and tear film instability before surgery. The height of lacrimal river in patients with exotropia and esotropia was 0.21 ± 0.06 mm and 0.17 ± 0.05 mm, and the mean BUT was <7 S. The mean conjunctival red eye index in patients with exotropia was 0.83 ± 0.37 [1]. With the increase of age, the conjunctival red eye index in patients with strabismus increased [1]. There were statistically significant differences in NIBUT and eye red index among multiple age groups in the concomitant exotropia [12]. A variety of tear proteins are significantly associated with aging in strabismus patients; many of which were associated with inflammation, immune response, and cell death. The persistence of inflammatory cytokines in ocular surface resulted in a series of pathophysiological changes, associated with abnormal of dry eye-related examines.

In this study, we selected several inflammatory cytokines associated with dry eye, including IL-17A, IL-10, IL-12p70, INF-*γ*, IL-6, and TNF-*α*. Dry eyes are a common ocular disease characterized by reduced tear production on both sides and unstable tear film. Goblet cell loss was directly related to surface cell apoptosis after chronic inflammatory injury, resulting in further tear film instability/imbalance [13]. IL-17A, a Th17-related cytokine, played an important role in the pathogenesis of dry eye disease, and its expression in tears may be a diagnostic biomarker for dry eye disease [14]. Significant differences in the concentration of cytokines (IL-6, IL-10, and TNF-*α*) were detected in tear samples collected from patients with dry eyes [15, 16]. Luminex technique indicated that the level of IL-12p70 in peripheral blood of patients with dry eye was significantly higher than that of normal control group [17]. Interferon-*γ* and IL-17A induced dry eye surface injury and the density of goblet cells in mice [18].

TNF-*α* and IL-6 are important factors of goblet cell apoptosis and dry eye symptoms in patients with concomitant exotropia. The production of TNF-*α* and IL-1 leads to the damage of dry eye surface in mice [18]. NF-*κ*B activation was an important regulator of TNF-*α* mediated apoptosis of corneal fibroblasts [19]. The activation of NF-*κ*B resulted in inflammation and apoptosis by on the ocular surface of dry eye, thereby reducing tear secretion [8]. TNF-*α* induces cell apoptosis by activating the p38 MAPK signaling pathway and acts as an effective TNF-*α* antagonist to prevent p38 MAPK-dependent apoptosis induced by TNF-*α* in cells [9].

The study has several limitations. Firstly, the number of samples included in this study was small, because we need to conduct rigorous screening of the subjects included in the study, and, it should be tested as soon as possible to prevent long-term storage of samples. Secondly, the experimental results of this study were not repeated for many times, and the test operation process was standardized by professional technicians without any information about sample type. Thirdly, some cytokine concentration in the research was lower than LLOQ, and we treated this part of data in accordance with the LLOQ of the corresponding panel, and this alternative result was reasonable.

## 5. Conclusion

In summary, we found that high expression levels of inflammatory factors (IL-6, and TNF-*α*) in tears of patients with concomitant exotropia were significant higher than the control, which could be a potential factor who promoted the occurrence of dry eye in the patients with concomitant exotropia. Further studies are needed to determine the ocular surface inflammation in patients with strabismus and whether the early intervention of ocular surface inflammation is beneficial to the prognosis of patients with concomitant strabismus.

## Figures and Tables

**Table 1:** The ocular surface analysis of included subjects.

	Number	Year	*p*	F/M	*p*
A. srabismus	36	28.11 ± 1.75		19 : 17	
A. control	36	31.56 ± 2.03	0.2029	19 : 17	>0.05
C. srabismus	30	9.80 ± 0.74		15 : 15	
C. control	30	8.77 ± 0.67	0.3041	15 : 15	>0.05

**Table 2:** The basic clinical information of the included subjects for tear analysis.

	Exotropia group (*n* = 11)	Control group (*n* = 11)	*p*
Gender (male : female)	6 : 5	6 : 5	>0.05
Age (year)	6 ± 0.4264	6 ± 0.4045	>0.05
Time (year)	1.25 ± 0.217	—	
Corneal light reflection test	−20.42 ± 1.9604	—	
Movement (unrestricted : restricted)	6 : 14	—	
Strabismus degree	−42.5Δ ± 3.5906	—	
The dominant eye (OD : OS)	6 : 5	—	
Symptoms (foreign-body sensation, pain, irritation, ocular fatigue, and eye redness)	2	—	

**Table 3:** The level of inflammatory cytokines in the tears.

	INF-*γ* (pg/mL)	IL-10 (pg/mL)	IL-12p70 (pg/mL)	IL-17A (pg/mL)	IL-6 (pg/mL)	TNF-*α* (pg/mL)
Exotropia	0.0284 ± 0.0125	0.3358 ± 0.1872	0.0253 ± 0.0078	0.1551 ± 0.0437	4.683 ± 1.329	0.2095 ± 0.0703
Control	0.0090 ± 0.0024	0.0513 ± 0.0149	0.0099 ± 0.0023	0.0793 ± 0.0313	1.455 ± 0.391	0.0513 ± 0.0149
*p*	0.1434	0.1506	0.0729	0.1739	0.0304	0.0397

## Data Availability

The data used to support the findings of this study are currently under embargo while the research findings are commercialized. Requests for data, 12 months after publication of this article, will be considered by the corresponding author.
